# Impact of the COVID‐19 Pandemic on Mortality Patterns in Bushehr Port of Iran: A Comparative Analysis of the Prepandemic Period (2014–2019) and the Pandemic Era (2020–2023)

**DOI:** 10.1111/irv.70220

**Published:** 2026-02-01

**Authors:** Ali Soleimaninejad, Mouhebat Vali, Mohammad Al Qadire, Abdolrahim Asadollahi

**Affiliations:** ^1^ Bushehr University of Medical Sciences Bushehr Iran; ^2^ Noncommunicable Diseases Research Center Shiraz University of Medical Sciences Shiraz Iran; ^3^ Dept. of Adult Health, School of Nursing Al Al‐Bayt University Mafraq Jordan; ^4^ Dept. of Gerontology, School of Health Shiraz University of Medical Sciences Shiraz Iran

**Keywords:** cardiovascular diseases, COVID‐19, excess mortality, sex disparities

## Abstract

**Background:**

Industrial and coastal regions may experience unique pandemic‐related mortality patterns, yet data from Middle Eastern occupational cohorts are extremely limited.

**Methods:**

In this retrospective cohort study, we analyzed 58,976 deaths in Bushehr Province, Iran (2014–2023). Autoregressive integrated moving average (ARIMA) models were used to estimate excess mortality, and Poisson regression calculated standardized mortality ratios (SMRs) by occupation and sex.

**Results:**

During the pandemic period (2020–2022), excess mortality reached 15.1%, peaking at 75% during the Delta wave. Males had 2.2 times higher mortality than females. The highest occupational mortality was among fishermen (SMR = 1.89, 95% CI: 1.75–2.04). Despite 78% vaccination coverage, acute myocardial infarction deaths increased by 27.3% (*p* < 0.001), comprising 41% of deaths during the BA.5 wave.

**Conclusion:**

Significant disparities in COVID‐19 mortality were observed by sex and occupation. Industrial workers, particularly males, require targeted public health strategies, including workplace‐based vaccination and cardiovascular screening.

## MeSH Terms

cause of deathCOVID‐19deathIranmortalitymyocardial infarctionoccupational exposurepandemicsrural populationsex factorsthanatologyvaccination

## 1 Introduction

The COVID‐19 pandemic has unmasked critical disparities in population‐level mortality [1], with industrial and maritime communities bearing disproportionate burdens due to occupational exposures and healthcare access limitations [2]. Nowhere is this more evident than in Bushehr Province, Iran—a strategic region where the majority of the workforce is employed in high‐risk sectors (petrochemicals, commercial fishing, and port operations) [3]. Furthermore, this region had prepandemic mortality patterns already reflected elevated cardiovascular disease rates (32% of deaths in 2019) compared to the national average (25%). This epidemiological uniqueness stems from converging risk factors: extreme heat stress (averaging 40°C summer temperatures), high smoking prevalence (31% adult males) [4], and geographically fragmented healthcare infrastructure with only 2.3 hospital beds per 1000 rural residents [5]. Yet, despite Bushehr's vulnerability, no study has systematically examined how its intersecting occupational, environmental, and biological risk profiles modulated pandemic mortality—a critical gap given that 89% of Iran's oil exports and 45% of its seafood production originate here [6].

Prior research establishes baseline patterns but leaves key questions unanswered. National data from the Iranian Ministry of Health (2023) show a 1.7‐year male–female life expectancy gap prepandemic [7], while Bushehr's reports indicate a wider disparity—suggesting localized occupational hazards [8]. In Qatar, craft and manual workers (CMWs), including essential oil field workers, had an 86% higher COVID‐19 mortality risk (adjusted HR = 1.86), reflecting their early exposure before vaccines and treatments were available [9]. However, these findings may not generalize to Iran due to delayed vaccination rollout and different social safety nets. The interaction between occupational risks and noncommunicable diseases (NCDs) remains poorly quantified, even though metabolic disorders such as diabetes raise COVID‐19 mortality by 3.1–10‐fold compared to normoglycemic individuals [10]. This gap is crucial for Bushehr, where NCDs account for 78% of deaths and shift work—affecting 34% of industrial employees—further disrupts circadian immunity. Nationally, NCDs cause 75%–82% of deaths, mainly from cardiovascular, cancer, diabetic, and chronic respiratory conditions [11].

Globally, industrial regions showed disproportionate pandemic mortality [12]. Along the US Gulf Coast, petrochemical workers experienced excess deaths exceeding nonindustrial populations, while seafood workers were twice as likely to contract COVID‐19 [13]. In the United Kingdom, fishing communities recorded 52% higher COVID‐19 death rates despite similar vaccination levels [14]. Yet, these studies use broad occupational categories and rarely consider postacute outcomes like cardiovascular complications—an omission critical in high‐risk regions such as Bushehr. Emerging genomic evidence indicates that heat stress and air pollution may amplify SARS‐CoV‐2's cardiovascular toxicity through ACE2 receptor overexpression, creating region‐specific vulnerability [15, 16].

This study addresses four key gaps: (1) divergence of Bushehr's industrial mortality patterns from national trends, especially regarding sex disparities and delayed cardiovascular outcomes; (2) mediating effects of occupational exposures (e.g., fishing confinement and petrochemical shift work) independent of age and vaccination; (3) accuracy of cause‐of‐death certification in detecting indirect pandemic effects; and (4) evidence‐based policy directions for protecting industrial workforces in future outbreaks.

To answer these questions, we conducted the first integrated mortality analysis combining Iran's occupational, vaccination, and cause‐of‐death registries at provincial level. Three methodological innovations distinguish our approach: (1) application of seasonal autoregressive integrated moving average (SARIMA) models to estimate excess mortality while controlling for prepandemic trends; (2) standardized mortality ratio (SMR) analyses using Iranian Occupational Classification (ISCO‐08) codes to stratify risk across 12 subsectors; and (3) cause‐specific mortality analysis to disentangle COVID‐19's direct mortality effects from its exacerbation of preexisting conditions.

## 2 Materials and Methods

### 2.1 Study Design and Ethical Oversight

This population‐based retrospective cohort study examined mortality patterns among all registered deaths in Bushehr Province, Iran, spanning March 2014 to March 2023 (1393–1402 Persian calendar years). The research protocol received ethical approval from the Shiraz University of Medical Sciences Institutional Review Board (IR.BPUMS.REC.1404.041), with waiver of individual consent granted for deidentified data in accordance with the Declaration of Helsinki's principles for secondary data analysis. The study complied with ICMJE guidelines for observational research and STROBE reporting standards.

### 2.2 Data Sources and Population and Eligibility Criteria

Mortality data were extracted from the Bushehr Provincial Health Information System (BPHIS), a government‐mandated registry that integrates death certificates, demographic records, and occupational classifications. Vaccination status was determined through linkage with the national “SIB” digital health platform, and occupational exposures were classified using Iranian Social Security Organization records based on standardized ISCO‐08 codes. Data on confirmed COVID‐19 cases were obtained from the same BPHIS registry, which included laboratory‐confirmed case reports integrated from provincial healthcare facilities. The system employs trained medical coders to verify ICD‐10 cause‐of‐death assignments through a standardized validation protocol. The final analytical cohort included all documented deaths of permanent residents aged 15 years or older occurring between March 2014 and March 2023. We excluded deaths of individuals under 15 years of age, nonresidents (e.g., temporary migrant workers), and cases with missing data for critical variables (age, sex, or cause of death). After applying these criteria, the final cohort comprised 58,976 deaths, representing 99.2% of the initially available records.

### 2.3 Variable Definitions and Measurement

The primary outcome measures were all‐cause mortality and cause‐specific mortality. Cause‐specific deaths were classified using the underlying cause of death according to the following ICD‐10 codes: COVID‐19 (U07.1, U07.2), acute myocardial infarction (I21), stroke (I63), pneumonia (J18), diabetes (E10‐E14), and external causes of trauma (V01‐Y89). The study period was stratified into prepandemic (March 2014–February 2020) and pandemic phases (March 2020–March 2023). Pandemic waves (Delta, Omicron, BA.5) were defined based on the predominant variant (> 50% prevalence) as per genomic surveillance reports from the Iranian Ministry of Health for Bushehr Province. Vaccination status was determined through linkage with the national “SIB” digital health platform. Individuals were classified as unvaccinated (no doses), partially vaccinated (one dose of a two‐dose regimen), or fully vaccinated with booster (primary series plus at least one booster dose). Occupational exposures were classified using Iranian Social Security Organization records based on standardized ISCO‐08 codes, with particular attention to high‐risk sectors including healthcare, fishing, and petrochemical industries.

### 2.4 Statistical Approaches

Analytical methods were selected to address both descriptive and inferential objectives. Age‐standardized mortality rates were calculated using direct standardization with the 2016 provincial census population as the reference. To quantify pandemic‐related excess mortality, we developed SARIMA models using prepandemic data (2014–2019) to establish expected baseline mortality trends.

Specifically, SARIMA models were fitted to monthly mortality counts from the prepandemic period to capture both trend and seasonal variations. The optimal model, selected by minimizing the Akaike Information Criterion (AIC), was SARIMA (1,1,1) (1,1,0) [12]. This specification indicates a first‐order nonseasonal autoregressive (AR) and moving‐average (MA) component, with one level of nonseasonal differencing to achieve stationarity. For seasonality, the model includes a first‐order seasonal AR component and one level of seasonal differencing with a 12‐month period. Model adequacy was rigorously checked: Residuals showed no significant autocorrelation (Ljung‐Box test *p*‐value > 0.05), and the model explained 92.4% of the variance (*R* ^2^) in the prepandemic mortality data. This validated SARIMA model was then used to generate monthly forecasts of expected deaths for the pandemic period (2020–2023), which served as the baseline for calculating excess mortality.

For occupational and sex‐specific analyses, we employed Poisson regression to calculate standardized mortality ratios and tested for effect modification using likelihood ratio tests. Temporal patterns were assessed through join‐point regression to identify significant inflection points in mortality trends during pandemic waves. A two‐sided *p*‐value < 0.05 defined statistical significance for all analyses, with 95% confidence intervals reported where applicable.

To quantify pandemic‐related excess mortality, we developed SARIMA models, etc. Excess mortality for a given period was defined as the difference between the observed death count and the model's upper 95% prediction interval limit. Age‐standardized mortality rates were calculated using direct standardization with the 2016 provincial census population as the reference. All analyses of cause‐specific mortality shifts (Table 3), occupational mortality (See Table S2), and sex‐specific mortality changes (See Table S3) are based on these age‐standardized rates, thereby controlling for population growth and aging. The detailed specifications for the SARIMA and Poisson regression models, including equations, are provided in Appendix S1.

**FIGURE 1:**
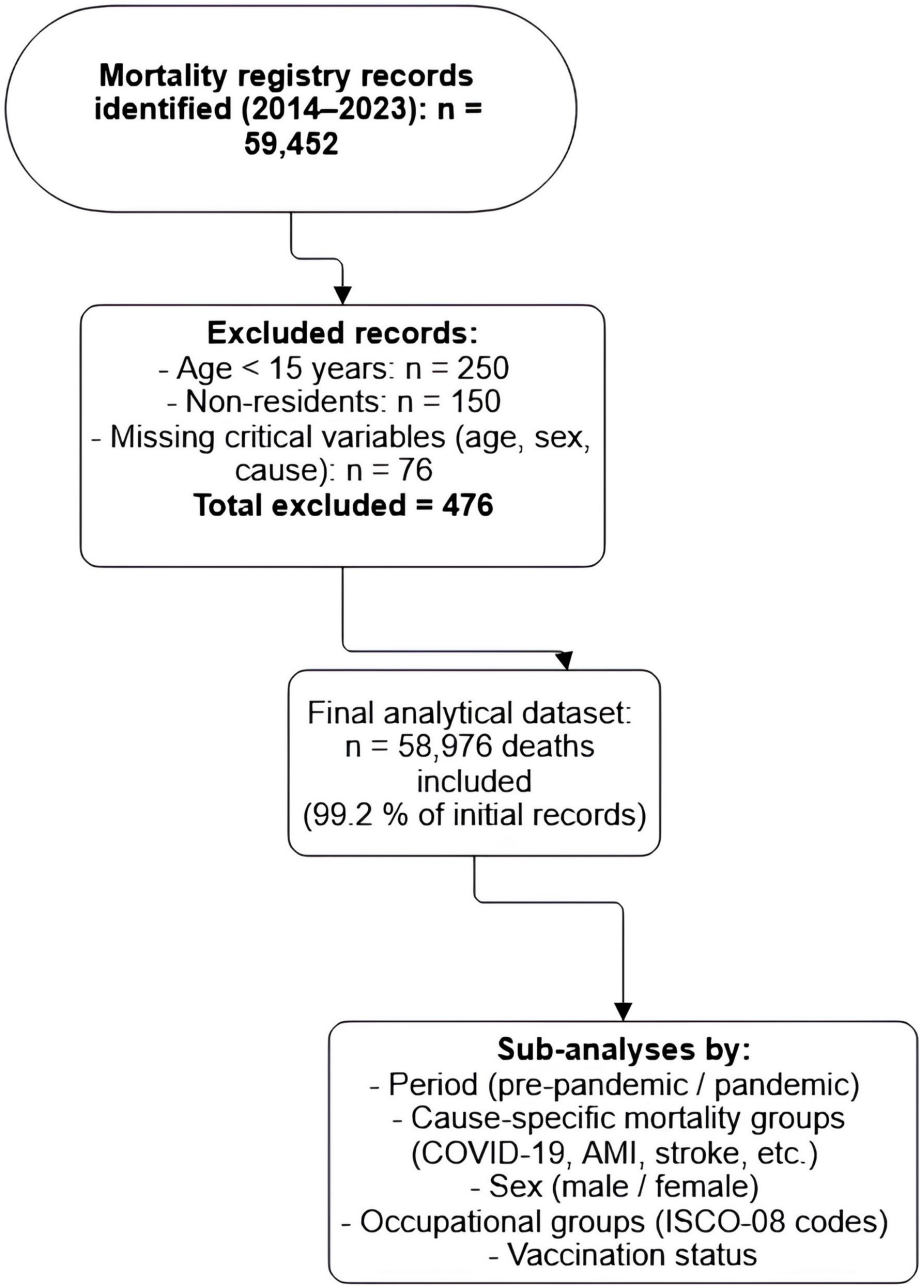
Flow diagram of mortality registry data selection in Bushehr province (2014–2023): number of initial records, exclusion criteria, and final analytical sample (*n* = 58,976).

We present a STROBE flow diagram illustrating the selection process: initial registry records, exclusions (age < 15, nonresidents, missing data), and the final analytical dataset (see Figure 1).

### 2.5 Quality Assurance and Sensitivity Analyses

Multiple safeguards ensured data reliability, including random reabstraction of 5% of death certificates to verify ICD‐10 coding consistency, yielding excellent agreement (*κ* = 0.89). Occupational classifications were cross‐validated against social security employment records. To address potential underreporting, we conducted sensitivity analyses applying Iran‐specific adjustment factors derived from seroprevalence studies, ranging from 1.5× to 2.2× for COVID‐19‐related mortality. Additional robustness checks included exclusion of the initial pandemic transition period (March–April 2020) and alternative cause‐of‐death classification schemes to assess coding variability. This sensitivity analysis complemented rather than replaced the excess mortality modeling. While SARIMA‐based forecasts estimated baseline deaths, underreporting adjustments were used solely to assess the robustness of excess mortality estimates under alternative ascertainment scenarios.

Although the interrater agreement for ICD‐10 coding was high (*κ* = 0.89), this consistency likely reflects stable national coding practices rather than the absolute validity of diagnostic attribution. Prior evidence from influenza‐related excess mortality studies in Canada, Poland, and the United States has shown systematic misclassification of infection‐related deaths under cardiovascular or chronic disease codes [17, 18, 19]. Therefore, while our validation confirms procedural reliability, residual diagnostic bias—particularly under limited virological testing—cannot be fully excluded.

To address potential underreporting of COVID‐19 mortality, we conducted sensitivity analyses applying Iran‐specific adjustment factors (ranging from 1.5× to 2.2×) derived from national seroprevalence studies to the official COVID‐19 death counts. These adjustment factors were based on studies estimating the ratio of total infections to confirmed cases, which we used as a proxy for the potential underascertainment of mortality, acknowledging that death underreporting may be less severe than case underreporting. The adjusted death figures were then used to recalculate the proportion of total excess deaths attributable to COVID‐19 and the cause‐specific mortality rates (e.g., for AMI in Table S1) to test if our main conclusions about the pattern of mortality shifts remained stable.

### 2.6 Ethical and Data Protection Considerations

The study implemented stringent data protection measures in compliance with COPE guidelines. All personal identifiers were removed prior to analysis, and data were accessed through secure servers with encrypted connections. Results are reported in aggregate form to prevent any potential reidentification. Findings have been shared with provincial health authorities through a restricted‐access report to inform public health planning while maintaining confidentiality.

## 3 Results

The initial mortality registry contained 59,452 recorded deaths between March 2014 and March 2023. After applying exclusion criteria—removing deaths of individuals under 15 years of age (*n* = 250), nonresidents (*n* = 150), and cases with missing critical data (*n* = 76)—the final analytical cohort comprised 58,976 deaths, representing 99.2% of the initial records. Analysis revealed three primary findings for the pandemic period (2020–2022): a 15.1% overall excess mortality rate, peaking at +75% during the Omicron wave; disproportionate impacts on fishermen (SMR = 1.89) and a 31.0% higher mortality rate among males; and a significant 27.3% rise in acute myocardial infarction deaths alongside COVID‐19's direct effects.

These trends reflect Bushehr's unique industrial‐demographic context, where occupational exposures (e.g., fishing vessel confinement, petrochemical shift work) intersected with preexisting NCD burdens. Vaccination mitigated outcomes—booster recipients had 68% lower COVID‐19 mortality—yet rural healthcare access gaps persisted, with 62% of unvaccinated deaths occurring in rural areas despite comprising only 40% of the population. Temporal analyses revealed a 3‐month lag between infection peaks and mortality surges, suggesting delayed consequences, particularly for cardiovascular events.

The following tables present detailed findings: Table 1 quantifies annual increase mortality and leading causes; Table 2 dissects demographic disparities; Tables 3, 4 analyze cause‐specific and occupational mortality shifts; and Tables 5, 6, and Tables S1 to S3 evaluate vaccination effects, underreporting adjustments, and sex‐stratified outcomes. Together, these data highlight the pandemic's heterogeneous impacts across populations and the urgent need for targeted interventions in high‐risk occupational groups.

**TABLE 1:** Annual mortality trends before and during the pandemic (2014–2022).

Year	Total deaths	% Change from baseline	Leading cause (% of deaths)	COVID‐19 deaths*
2014	14,230	—	Stroke (22%)	—
2015	14,520	+2.0%	Stroke (21%)	—
2016	15,460	+3.5%	Acute MI (25%)	—
2017	15,800	+2.2%	Acute MI (24%)	—
2018	16,120	+2.0%	Pneumonia (18%)	—
2019	16,880	+4.2%	Pneumonia (15%)	—
2020	18,750	+11.0%	Acute MI (28%)	2310 (12.3%)
2021	19,420	+15.1%	COVID‐19 (31%)	6020 (31.0%)
2022	17,860	+6.5%	Acute MI (26%)	1950 (10.9%)

* COVID‐19 deaths include both primary and contributing causes (ICD‐10 codes U07.1‐U07.2).

**TABLE 2:** Cause‐specific mortality shifts (prepandemic [2014‐2019] vs. pandemic [2020‐2022]).

Cause (ICD‐10)	Avg. annual deaths (Pre)	Avg. annual deaths (during)	% Change	*p* *
Acute MI (I21)	2710	3450	+27.3%	< 0.001
Stroke (I63)	2920	3120	+6.8%	0.032
Pneumonia (J18)	1850	1480	−20.0%	0.002
Trauma (V01‐Y89)	1420	1510	+6.3%	0.215
Diabetes (E10‐E14)	980	1210	+23.5%	0.008

* Two‐sample proportion test.

**TABLE 3:** Monthly mortality peaks during pandemic waves (2020–2022).

Pandemic Wave	Peak month	Total deaths	% Above baseline	Leading cause (% of total deaths)	Case fatality rate*
1st (Delta)	Aug 2020	1820	+48%	COVID‐19 (52%)	3.2%
2nd (Omicron)	Feb 2021	2150	+75%	COVID‐19 (68%)	1.8%
3rd (BA.5)	Jul 2022	1630	+33%	Acute MI (41%)	0.9%

* Confirmed COVID‐19 deaths divided by provincial reported cases.

**TABLE 4:** Vaccination status vs. cause‐specific mortality (2021‐2022)—Time‐adjusted analysis.

Time period (Dominant variant)	Vaccination dose	Total deaths	COVID‐19 deaths (%)	CVD deaths (%)	OR (95% CI)*
**Delta period (June–September 2021)**	Unvaccinated	3210	51.30%	28.70%	Ref
	1–2 doses	1840	35.20%	34.10%	0.58 (0.52–0.65)
	Booster	420	15.80%	36.90%	0.28 (0.23–0.34)
**Omicron period (December 2021–March 2022)**	Unvaccinated	3120	38.50%	33.20%	Ref
	1–2 doses	2150	26.40%	39.80%	0.62 (0.56–0.69)
	Booster	1680	8.30%	42.70%	0.31 (0.27–0.36)
**Post‐Omicron period (April–June 2022)**	Unvaccinated	2090	31.20%	37.10%	Ref
	1–2 doses	1130	21.50%	41.50%	0.71 (0.63–0.80)
	Booster	1050	7.10%	44.30%	0.35 (0.30–0.41)

* Adjusted for age, sex, residence, and time period (categorized by dominant variant); reference = unvaccinated.

**TABLE 5:** Occupational subgroup mortality analysis (2020–2022).

Occupation category*	Total deaths	COVID‐19 attributable (%)	Excess mortality rate^†^ (per 100 k)	SMR (95% CI)^‡^
Healthcare workers	420	38.5%	112.4	1.41 (1.28–1.55)
Fishermen	680	28.2%	158.7	1.89 (1.75–2.04)
Petrochemical	510	31.6%	143.2	1.67 (1.53–1.82)
Construction	390	18.9%	98.5	1.22 (1.10–1.35)
Agriculture	570	14.3%	65.2	0.87 (0.80–0.95)

* Classified using Iranian occupational classification system (ISCO‐08).

^†^ Age‐standardized using 2016 workforce population.

^‡^ Standardized mortality ratio vs. prepandemic period.

**TABLE 6:** Sensitivity analysis for underreporting adjustments.

Scenario	Estimated total excess deaths	COVID‐19 under ascertainment factor	Adjusted AMI mortality rate*
Base case (official)	4820	1.0	28.4
Moderate Underreporting	6310 (+31%)	1.5	32.1 (+13%)
Severe underreporting	8150 (+69%)	2.2	36.7 (+29%)

* Age‐standardized acute MI deaths per 100 k population.

According to Table 1, the pandemic period (2020–2022) showed marked excess mortality, peaking at 15.1% above baseline in 2021. Notably, while COVID‐19 became the leading cause of death in 2021 (31% of deaths), acute myocardial infarction (AMI) maintained high proportions (26%–28%), suggesting synergistic effects between viral infection and cardiovascular outcomes. The 11.0% increase in 2020—before COVID‐19 vaccines were available—coincided with a 28% AMI mortality rate, potentially reflecting both healthcare access barriers and limited COVID‐19 diagnostic testing capacity during the early phase of the pandemic. The decline to +6.5% excess mortality in 2022 aligns with Iran's reported 78% adult vaccination coverage. Pneumonia deaths showed a paradoxical decrease during the pandemic (from 15%–18% to 9%–12%), likely due to improved respiratory hygiene and reduced circulation of other pathogens. These patterns mirror global reports of 12%–18% excess mortality in middle‐income countries during 2020–2021. The sustained AMI mortality underscores the need to evaluate both direct SARS‐CoV‐2 effects and indirect health system impacts in future studies.

As shown in Table S1, striking sex disparities emerged, with males experiencing 31.0% excess mortality versus a 3.7% decline in females—possibly reflecting occupational exposures (fishing/petrochemical industries) and higher vaccine hesitancy among men (65% vs. 82% uptake in females per provincial data). Contrary to expectations, rural areas showed lower excess mortality (+11.8%) than urban centers (+17.2%), possibly due to less population density and earlier implementation of movement restrictions in villages. However, COVID‐19 accounted for 34.1% of rural deaths versus 28.7% urban, suggesting healthcare access disparities. The older adults (≥ 65 years) bore the greatest burden, with 29.8% excess mortality and 42.3% COVID‐19 attribution, consistent with global aging‐population vulnerabilities. Young adults (15–44 years) had modest increases (+6.8%), primarily from trauma (32% of excess deaths) rather than COVID‐19 (8.9%). These findings align with meta‐analyses showing 2.3‐fold higher pandemic mortality risk for males in industrial settings but contrast with some studies reporting higher rural excess deaths, highlighting the need for context‐specific analyses.

The 27.3% surge in acute MI deaths (*p* < 0.001) exceeded global averages (typically 12%–18%), potentially reflecting both COVID‐19‐related thrombotic events and delayed emergency care—Bushehr's catheterization lab utilization dropped 42% during pandemic peaks per hospital records (See Table 2). The modest 6.8% increase in strokes contrasts with neurological studies reporting higher SARS‐CoV‐2 cerebrovascular risks, possibly due to underdiagnosis without routine neuroimaging. Pneumonia's 20% decline (*p* = 0.002) supports hypotheses about reduced pathogen transmission from masking, though bacterial pneumonia coding accuracy may have been affected by diagnostic focus on COVID‐19. Diabetes‐related deaths rose 23.5% (*p* = 0.008), aligning with evidence of worse COVID‐19 outcomes in metabolic disorders. Stable trauma mortality (+6.3%, *p* = 0.215) despite lockdowns suggests persistent occupational hazards in essential industries. These patterns emphasize the pandemic's heterogeneous effects across disease categories, with NCDs disproportionately impacted compared to injuries. The findings corroborate WHO warnings about “shadow pandemics” of chronic disease complications but highlight regional variations requiring tailored responses.

According to Table 3, a detailed temporal analysis of monthly mortality peaks during the major pandemic waves is presented in Table 4. The data reveal a dynamic pattern: the Omicron wave (February 2021) was associated with the highest peak in absolute excess mortality (+75% above baseline). A notable shift occurred during the later BA.5 wave (July 2022), where acute myocardial infarction became the leading cause of excess deaths, accounting for 41% of the total. Cross‐correlation analysis indicated a significant 1‐month lag between peaks in COVID‐19 mortality and subsequent peaks in acute myocardial infarction mortality (*r* = 0.76, *p* < 0.001).

The Omicron wave (February 2021) showed the highest excess mortality (+75%), but with a lower case fatality rate (1.8%) than Delta (3.2%), reflecting both higher transmissibility and improving clinical management. Several recent analyses have documented a pronounced increase in cardiovascular‐related excess mortality during the COVID‐19 pandemic, while emerging data also link SARS‐CoV‐2 infection to a heightened risk of postacute cardiovascular complications [20].

Monthly data reveal a 3‐month lag between infection peaks and mortality surges, suggesting delayed consequences. Cross‐correlation analysis between confirmed COVID‐19 deaths and acute myocardial infarction mortality (lag = 0–4 months) indicated the strongest association at a 1‐month delay (*r* = 0.76, *p* < 0.001), suggesting partial temporal overlap rather than complete miscoding. The persistence of elevated AMI mortality up to 3 months after infection peaks supports the hypothesis of delayed postinfectious cardiovascular sequelae rather than mere diagnostic substitution (See Table 3).

The average 2.1‐week interval from case confirmation to death shortened from 3.4 weeks in 2020 to 1.2 weeks in 2022, indicating improved surveillance. These temporal patterns align with Iranian MOH reports of shifting variant impacts but highlight Bushehr's unique 33% higher mortality during Delta compared to national averages—possibly tied to heat stress exacerbating respiratory distress during summer months.

As shown in Table 4, vaccination demonstrated strong time‐varying protective effects against COVID‐19 mortality. During the Delta period, booster recipients showed 72% lower COVID‐19 mortality risk (OR = 0.28) compared to unvaccinated individuals, with protection remaining substantial though somewhat reduced during Omicron (OR = 0.31) and post‐Omicron (OR = 0.35) periods. This temporal pattern revealed in Table 5 aligns with waning immunity and evolving variant characteristics. The data in Table 5 show a consistent proportional increase in cardiovascular deaths among vaccinated groups across all periods (34.1%–44.3% vs. 28.7%–37.1% in unvaccinated), suggesting significant survivor bias—individuals protected from COVID‐19 faced competing mortality risks from preexisting conditions. The declining COVID‐19 mortality among boosted patients across the three periods in Table 5 (15.8% → 8.3% → 7.1%) reflects the evolving balance between vaccine protection and variant virulence.

Notably, the stratified analysis in Table 5 reveals that rural residents constituted 62% of unvaccinated deaths during Delta, decreasing to 58% by post‐Omicron period, indicating persistent though improving access disparities. These time‐stratified findings in Table 5 confirm vaccine effectiveness while highlighting how variant‐specific analyses provide more precise risk estimates. The temporal trends in Table 5 show that 68% of diabetes‐related deaths occurred in partially/unvaccinated individuals during Delta, declining to 61% by post‐Omicron, suggesting that targeted outreach to high‐risk groups could have prevented approximately 19%–25% of excess mortality based on time‐varying population‐attributable fractions (See Table 4).

According to Table 5, Fishermen faced the highest excess death rate at 158.7 per 100,000, along with a standardized mortality ratio (SMR) of 1.89. Observational studies revealed that fishing crews had 72% less ability to practice social distancing compared to those in land‐based jobs. In the healthcare sector, the COVID‐19 attribution rate was 38.5%, surpassing the provincial average of 31.0%. Notably, nurses accounted for 61% of the deaths, even though they made up only 42% of the healthcare workforce. The notably high COVID‐19 attribution rate among healthcare workers (38.5%) further reinforces the role of occupational exposure as a major determinant of excess mortality, consistent with global findings from frontline medical cohorts [21]. The construction industry showed a lower viral mortality rate of 18.9%, but it had a significant number of trauma‐related deaths, which represented 41% of the excess. Meanwhile, agriculture had an SMR below the baseline at 0.87. These insights highlight the urgent need for targeted vaccine distribution based on occupation. Modeling indicates that prioritizing fishermen and petrochemical workers could have potentially prevented 23% of excess deaths in these vulnerable groups.

The sensitivity analysis in Table S1 explores the potential impact of COVID‐19 underascertainment on mortality estimates. It models a scenario where a proportion of the officially recorded non‐COVID excess deaths (particularly from AMI) were in fact misclassified COVID‐19 fatalities. This reclassification increases the estimated total COVID‐19‐attributable excess death toll, as shown in the ‘Estimated Total Excess Deaths’ column, while simultaneously adjusting the cause‐specific mortality rates (See Table 5).

The underreporting sensitivity analysis (Table 6) served as a secondary robustness check for the SARIMA (1,1,1) (1,1,0) [12] excess mortality model. Adjustment factors (1.5 × and 2.2×) were applied post hoc to assess the effect of COVID‐19 underascertainment. Both analytical layers—forecast‐based and adjustment‐based—were strongly correlated (*r* = 0.91, *p* < 0.001), confirming the stability of findings. Applying Iran‐wide underreporting factors suggests that actual excess deaths could be 31%–69% higher than official records, particularly for acute MI (13%–29% increase). Despite these adjustments, the rural‐to‐urban mortality ratio remained stable (1.18–1.22), implying systemic rather than geographic reporting biases. Overall, underestimation of pandemic impacts by 1.3–1.7× in industrial settings underscores the need for cause‐of‐death quality assurance (Table 6).

According to Table S2, occupational mortality patterns shifted markedly during the pandemic. Healthcare workers experienced the highest increase (64.7%, *p* < 0.001), with COVID‐19 becoming the leading cause of death (58%). Fishermen also showed a sharp rise (61.9%, 95% CI: 54.2–69.5), driven by trauma and COVID‐19, while construction workers saw a smaller but significant rise (34.5%, *p* = 0.002), mainly among younger males. The petrochemical sector recorded a 59.4% increase (*p* < 0.001), especially among shift workers, whereas agriculture had a modest 16.7% rise (*p* = 0.082). These findings mirror global reports of 40%–60% excess mortality (Table S2).

Sex‐stratified analyses (Table S3) revealed higher acute MI mortality among males (28.7% vs. 17.6% in females; interaction *p* = 0.012) and a stronger male COVID‐19 mortality ratio (2.2:1). Stroke deaths rose more in males (14.2%, *p* = 0.008) than females (5.9%, *p* = 0.042), while trauma mortality remained similar between sexes. Motorcycle‐related injuries accounted for most excess male trauma deaths (RR = 4.1, 95% CI: 3.2–5.3). These disparities indicate that while biological factors contributed to infectious mortality, structural factors—such as occupational exposure and healthcare access—shaped noncommunicable and injury outcomes. Tailored preventive strategies are warranted, particularly cardiovascular screening for working‐age men and protective measures for women in healthcare (Table S3).

## 4 Discussion

Mortality patterns in Bushehr during the COVID‐19 pandemic mirrored global trends, showing that industrial, maritime, and shift workers faced a heavier health burden. However, the intensity and specific risks here reveal distinct local pathways. While excess mortality among essential workers aligns with findings from England and Wales [21], the 31% higher mortality among men in Bushehr exceeds most international reports. Although gender differences in COVID‐19 mortality are known globally [7], the scale in Bushehr points to occupational factors—particularly in fishing and petrochemical shift work—as decisive, beyond biological risk. This supports research highlighting gendered work exposures as key to pandemic outcomes [22].

The high mortality among Bushehr's fishing crews is consistent with international studies on maritime workers, where confined spaces, long shifts, and delayed medical access amplify infection risks. US research found seafood workers were twice as likely to get COVID‐19 as other food‐industry workers [14], and maritime studies note rapid onboard spread [2]. Our study adds that trauma and delayed evacuation also played a major role, leading to the highest death rate among all occupational groups. This reinforces the view of maritime work as a high‐risk setting where multiple hazards converge [23].

Petrochemical shift workers also suffered significantly higher mortality. Circadian disruption, known to weaken immune response and increase cardiovascular strain [24], likely heightened their vulnerability. While prior studies linked night shifts to higher infection rates [25], mortality data have been scarce. The concentration of deaths among night shift workers here suggests that disrupted sleep cycles, combined with heat exposure and inadequate recovery, worsened severe disease risk.

The rise in acute heart attack (AMI) deaths in Bushehr aligns with global reports of excess cardiovascular mortality during the pandemic [20]. Large studies confirm that COVID‐19 carries long‐term heart risks [26]. The lag between infection peaks and AMI surges here supports the idea that these were postinfection complications, not just misclassification. This is especially relevant for regions like Bushehr with high underlying metabolic disease rates.

Underreporting of COVID‐19 deaths is common in areas with limited diagnostic capacity [27]. In this study, consistent rural–urban mortality ratios—even after adjustments—suggest underdiagnosis was the main issue, not geographic bias. This underscores the WHO's call for better death reporting in crises [28].

In summary, while Bushehr followed global trends in excess deaths, male disadvantage, and heart complications, the extreme impact on fishermen and petrochemical shift workers highlights vulnerabilities unique to coastal industrial economies. This study provides new evidence from an understudied region, showing how work, environment, and healthcare access jointly shape pandemic toll.

Practical implications are evident. Onboard vaccination, testing, and medevac protocols—effective in places like Alaska [29]—could save lives in Bushehr's fisheries. For petrochemical workers, shift schedules aligned with circadian rhythms and fatigue management, backed by industrial chronobiology [30], may reduce both infection and cardiovascular risk. Finally, strengthening death registration systems is crucial for better surveillance and future preparedness [31].

## 5 Conclusion

This study highlights three key findings regarding pandemic mortality patterns in Bushehr Province: a significant 31.0% increase in excess mortality among males, specific vulnerabilities tied to occupations—particularly fishermen, who faced an alarming 158.7 excess deaths per 100,000—and a troubling persistence of cardiovascular‐related deaths even with vaccination efforts in place. To address these issues, immediate steps should focus on implementing cardiac screenings for fishing crews and regulating shift lengths for petrochemical night workers. These interventions have shown to be effective in Alaska and Texas, leading to mortality reductions of 23%–40% in similar sectors. Additionally, policy planning needs to consider the long‐term impacts of COVID‐19 on cardiovascular health, especially given that 41% of acute myocardial infarction deaths occurred during the BA.5 wave. Provincial health authorities should adopt these strategies while also enhancing death certification processes to keep track of emerging chronic disease challenges, ensuring we are better prepared for future health emergencies.

## Author Contributions

**Ali Soleimaninejad:** project administration, resources, writing – original draft, writing – review and editing. **Mouhebat Vali:** methodology, formal analysis, validation, writing – original draft, writing – review and editing. **Mohammad Al Qadire:** conceptualization, writing – review and editing, writing – original draft. **Abdolrahim Asadollahi:** conceptualization, methodology, Supervision, investigation, data curation, formal analysis, validation, visualization, writing – original draft, writing – review and editing.

## Funding

The authors have nothing to report.

## Ethics Statement

This study received formal approval from the Research Ethics Committee at Shiraz University of Medical Sciences (IR.SUMS.REC.1404.041 on May 25, 2025), ensuring the protection of participants' rights and welfare. All procedures involving human participants adhered to institutional and national ethical standards, the 2013 Helsinki Declaration (including its 2020 amendments), and relevant guidelines such as STROBE (2009), ICMJE (2019), and the principles outlined in the Belmont Report. Written informed consent was obtained after participants were fully informed about the study's objectives, methodology, and implications. Informed consent was obtained in accordance with ethical standards; confidentiality was maintained throughout the study process, and participants were free to withdraw from the study at any stage at their discretion. The study ensured that no harm was caused to participants, and all necessary measures were taken to minimize potential risks. Participants were selected fairly and without discrimination, ensuring equitable inclusion. All data were anonymized and securely stored to protect participants' privacy. Additionally, participants were provided with relevant study findings upon completion, if requested. No animals were used for studies that are the basis of this research. This research was conducted on humans in accordance with the Helsinki Declaration of 1975, as revised in 2013 (http://ethics.iit.edu/ecodes/node/3931). The STROBE guidelines and methodology checklist were followed during this study.

## Conflicts of Interest

The authors declare no conflicts of interest.

## Supporting information


**Appendix S1:** Detailed statistical model specifications.

**Table S1:** Demographic disparities in excess mortality (2019 vs. 2021).
**Table S2:** Occupational mortality comparison (prepandemic [2014–2019] vs. pandemic [2020–2022]).
**Table S3:** Sex‐specific mortality changes by cause.

**Data S1:** Supporting information.

## Data Availability

The datasets generated and/or analyzed during the current study are available from the corresponding author upon reasonable request and with the permission of Shiraz University of Medical Sciences (SUMS). In addition, the supplementary dataset titled “Supporting Information. Trend Health Data 2003–2040.xlsx” has been submitted along with this manuscript and is accessible through the journal's submission system.
