# Supplementary material for: Impact of the COVID‐19 Pandemic on Mortality Patterns in Bushehr Port of Iran: A Comparative Analysis of the Prepandemic Period (2014–2019) and the Pandemic Era (2020–2023)
Source: Influenza Other Respir Viruses. 2026 Feb 1;20(2):e70220. doi: 10.1111/irv.70220 (PMC12862235; doi:10.1111/irv.70220)
Supplement: Supplementary file 1 — Appendix S1: Detailed statistical model specifications. [file IRV-20-e70220-s001.docx]

**Supplementary Appendix S1. Detailed Statistical Model Specifications**

**1. SARIMA Model Formulation**
The Seasonal ARIMA (SARIMA) model is denoted as SARIMA(p,d,q)(P,D,Q)[s] and defined by the equation:

(1 - φ₁B - ... - φₚBᵖ)(1 - Φ₁Bˢ - ... - ΦₚBᵖˢ) (1 - B)ᵈ(1 - Bˢ)ᵈ Yₜ = (1 + θ₁B + ... + θₚBᵖ)(1 + Θ₁Bˢ + ... + ΘₚBᵖˢ) εₜ

Where:

- Yₜ is the monthly death count at time t.
- B is the backshift operator (BYₜ = Yₜ₋₁).
- φᵢ (non-seasonal AR), θᵢ (non-seasonal MA), Φᵢ (seasonal AR), Θᵢ (seasonal MA) are parameters.
- d and D are degrees of non-seasonal and seasonal differencing.
- s is the seasonal period (s=12 for monthly data).
- εₜ is the white noise error term.

Our final model was SARIMA(1,1,1)(1,1,0)[12].

**2. Poisson Regression Model for SMRs**
The Poisson model for calculating Standardized Mortality Ratios (SMRs) was:
log(E[Deathsᵢ]) = log(Populationᵢ) + β₀ + β₁(Period) + β₂(Occupation) + ...
Where the SMR is exp(β) for the coefficient of interest, adjusted for other model terms.

**3. Calculation of Cause-Specific Mortality Shifts (Table 3)**
The percent change was calculated using age-standardized mortality rates (ASMRs):
% Change = [(ASMR_during - ASMR_pre) / ASMR_pre] * 100

**4. Calculation of Occupational Mortality (See Supplementary Table 2)**
The percent change in average annual deaths was calculated. The significance test (McNemar) assessed the change in the distribution of underlying causes of death between periods.

**5. Calculation of Sex-Specific Mortality Changes (Supplementary Table 3)**
The percent change (%Δ) for each sex and cause was calculated from pre-pandemic to pandemic periods using age-standardized rates. The interaction p-value (Breslow-Day test) assessed whether the mortality change differed significantly between males and females.

------------------------------------------------------------------------------------------
