# Supplementary material for: Impact of the COVID‐19 Pandemic on Mortality Patterns in Bushehr Port of Iran: A Comparative Analysis of the Prepandemic Period (2014–2019) and the Pandemic Era (2020–2023)
Source: Influenza Other Respir Viruses. 2026 Feb 1;20(2):e70220. doi: 10.1111/irv.70220 (PMC12862235; doi:10.1111/irv.70220)
Supplement: Supplementary file 2 — Table S1: Demographic disparities in excess mortality (2019 vs. 2021). Table S2: Occupational mortality comparison (prepandemic [2014–2019] vs. pandemic [2020–2022]). Table S3: Sex‐specific mortality changes by cause. [file IRV-20-e70220-s003.docx]

**Supplementary Appendix S2: Tables**

**Supplementary Table 1.** Demographic Disparities in Excess Mortality (2019 vs. 2021)

| **Group** | **2019 Deaths** | **2021 Deaths** | **% Increase** | **COVID-19 Attributable (%)** |
| --- | --- | --- | --- | --- |
| Male | 9,120 | 11,950 | +31.0% | 38.2% |
| Female | 7,760 | 7,470 | -3.7% | 19.5% |
| Urban | 10,120 | 11,860 | +17.2% | 28.7% |
| Rural | 6,760 | 7,560 | +11.8% | 34.1% |
| Age ≥65 | 10,980 | 14,250 | +29.8% | 42.3% |
| Age 15-44 | 1,320 | 1,410 | +6.8% | 8.9% |

**Supplementary Table 2.** Occupational Mortality Comparison (Pre-Pandemic [2014-2019] vs. Pandemic [2020-2022])

| **Occupation** | **Avg. Annual Deaths (Pre)** | **Avg. Annual Deaths (During)** | **% Change** | **Leading Cause Shift*** | **Gender Distribution (M:F)†** |
| --- | --- | --- | --- | --- | --- |
| Healthcare Workers | 85 | 140 (+64.7%) | <0.001 | Respiratory → COVID-19 (p<0.001) | 1 : 2.1 → 1 : 3.4 |
| Fishing | 210 | 340 (+61.9%) | <0.001 | Trauma → Mixed (COVID-19 + Trauma) | 5.8 : 1 → 6.3 : 1 |
| Petrochemical | 160 | 255 (+59.4%) | <0.001 | CVD → COVID-19 (p=0.003) | 9.2 : 1 → 8.7 : 1 |
| Construction | 145 | 195 (+34.5%) | 0.002 | Trauma (stable) | 12.4 : 1 → 11.9 : 1 |
| Agriculture | 180 | 210 (+16.7%) | 0.082 | CVD → Respiratory (p=0.041) | 3.1 : 1 → 2.8 : 1 |
| **McNemar test for cause-of-death distribution changes*  *†Male to Female ratio shown for pre-pandemic → pandemic periods* | | | | | |

**Supplementary Table 3.** Sex-Specific Mortality Changes by Cause

| **Cause (ICD-10)** | **Male Deaths (Pre)** | **Male Deaths (During)** | **%Δ** | **Female Deaths (Pre)** | **Female Deaths (During)** | **%Δ** | **Interaction p-value**** |
| --- | --- | --- | --- | --- | --- | --- | --- |
| COVID-19 (U07) | - | 4,120 | - | - | 1,880 | - | - |
| Acute MI (I21) | 1,950 | 2,510 | +28.7% | 760 | 890 | +17.6% | 0.012 |
| Stroke (I63) | 1,620 | 1,850 | +14.2% | 680 | 720 | +5.9% | 0.043 |
| Trauma (V01-Y89) | 1,150 | 1,240 | +7.8% | 270 | 290 | +7.4% | 0.891 |

***Breslow-Day test for homogeneity of odds ratios.*
